# Dental development in *Homo naledi*

**DOI:** 10.1098/rsbl.2017.0339

**Published:** 2017-08-30

**Authors:** Zachary Cofran, Christopher S. Walker

**Affiliations:** 1 Anthropology Department, Vassar College, Poughkeepsie, NY, USA; 2 Department of Molecular Biomedical Sciences, College of Veterinary Medicine, North Carolina State University, Raleigh, NC, USA; 3 Department of Evolutionary Anthropology, Duke University, Durham, NC, USA; 4 Evolutionary Studies Institute and Centre for Excellence in PalaeoSciences, University of the Witwatersrand, Johannesburg-Braamfontein, Gauteng, South Africa

**Keywords:** hominin, teeth, tooth eruption, life history, ontogeny

## Abstract

Humans’ prolonged somatic development and life history are unique among primates, yet their evolutionary origins remain unclear. Dental development has been used as a proxy to reconstruct life history evolution in the hominin clade and indicates a recent emergence of the human developmental pattern. Here, we analyse tooth formation and eruption in two developing dentitions of *Homo naledi*, a late-surviving, morphologically mosaic hominin species. Deciduous dental development is more similar to humans than to chimpanzees, probably reflecting hominin symplesiomorphy rather than bearing life history significance. The later stages of permanent tooth development present a mix of human- and chimpanzee-like patterns. Surprisingly, the M_2_ of *H. naledi* emerges late in the eruption sequence, a pattern previously unknown in fossil hominins and common in modern humans. This pattern has been argued to reflect a slow life history and is unexpected in a small-brained hominin. The geological age of *H. naledi* (approx. 300 kya), coupled with its small brain size and the dental development data presented here, raise questions about the relationship between dental development and other variables associated with life history.

## 1. Introduction

Patterns and sequences of dental development are intimately connected to species' life history. Schultz noted that primates differ from many other mammals in having relatively accelerated replacement of the deciduous teeth and delayed addition of the permanent molars [1,2]. As humans are the most extreme example of this pattern, with the permanent molars M_2–3_ usually emerging last of all, he linked humans’ tooth emergence sequence with our prolonged period of growth. In the contexts of hominin evolution, dental development has been a key source of inference about life history of extinct species. Members of *Australopithecus* and early *Homo* generally display faster permanent tooth crown formation and earlier ages of tooth emergence than is observed in recent humans [3–6], and their greater conformity to chimpanzee patterns of dental development suggests that slow life history appeared relatively late in human evolution.

**Figure 1.**
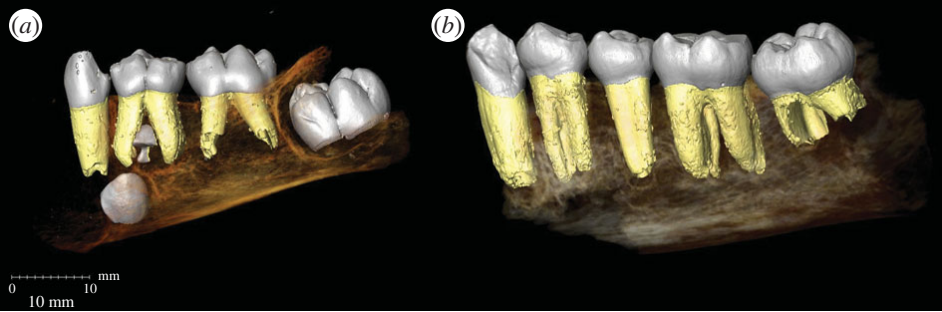
Tooth emergence and formation in U.W. 101-1400 (*a*) and U.W. 101-377 (*b*). (Online version in colour.)

The recently discovered species *H. naledi* displays many primitive features (e.g. australopith-sized brain [7]) at an unexpectedly recent date (236–335 kya [8]). This presents a unique opportunity to evaluate life history patterns in a primitive hominin that temporally overlapped with much larger-brained *Homo* species, and to elucidate our understanding of life history evolution in the human lineage. Two immature mandibles with securely associated dentitions allow reconstruction of dental development in *H. naledi* (figure 1; electronic supplementary material, text S1), which we compare to dental ontogeny in chimpanzees (*Pan troglodytes*) and hominins.

## 2. Material and methods

**Figure 2.**
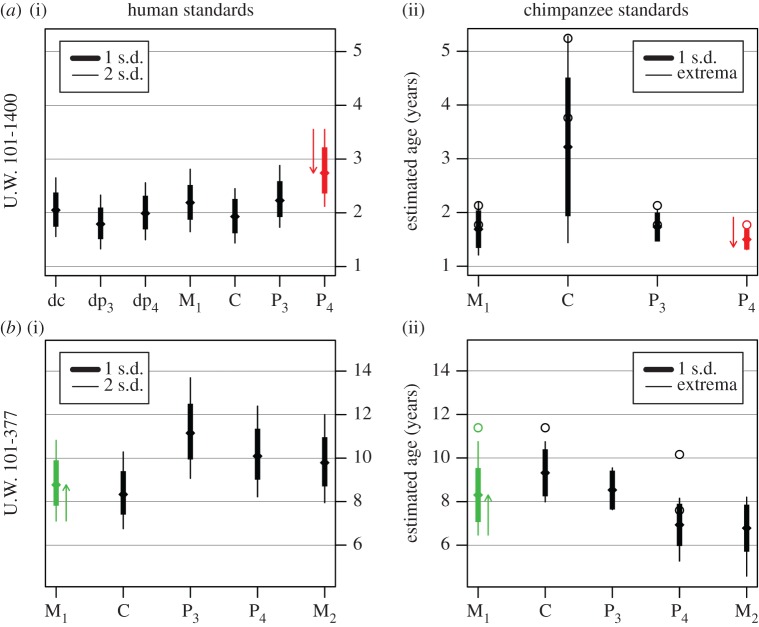
Estimated age at death from each tooth of U.W. 101-1400 (*a*) and 101-377 (*b*), based on human (i) and chimpanzee (ii) formation standards. Tick marks indicate mean estimates, thick lines extend to ±1 s.d., and thin lines extend to ±2 s.d. (humans) or sample extremes (chimpanzees). Open circles represent wild chimpanzees. Red bars indicate initiation of the P_4_ that had not begun in U.W. 101-1400. Green bars indicate minimum age estimates for the M_1_ that had completed formation in U.W. 101-377. Note deciduous formation standards are not available for chimpanzees. (Online version in colour.)

Tooth emergence was observed on original specimens. Tooth formation was assessed from microCT scans, produced at the University of Witwatersrand microCT facility on a Nikon Metrology XTH 225/320 LC dual-source scanner. Scans were conducted with 95 kV energy and 95 µA current, with an isometric voxel size of 30.8 µm. CT images were processed in Avizo 6.3 (VSG, Burlington MA, USA). Developing crown heights and root lengths were measured from CT images with ImageJ (http://rsb.info.nih.gov/ij/), and from three-dimensional surface renderings in Geomagic Studio (www.geomagic.com). Stages of crown and root formation were scored according to published standards for comparison with other studies [9–13] (electronic supplementary material, text S2 and table S3). To determine whether the overall pattern of tooth formation is more similar to humans or chimpanzees, we used these standards to obtain age estimates of individual teeth for each specimen (figure 2). The purpose is not to estimate the specimens' chronological ages, but to see whether all of an individual's teeth provide the same signal (e.g. if *H. naledi* followed a human-like pattern then each tooth should produce similar age estimates by human standards, but different estimates by chimpanzee standards).

## 3. Results

### (a) U.W. 101-1400

Tooth wear indicates a deciduous emergence sequence of [di_1_–di_2_]–dp_3_–dc–dp_4_ (brackets mean the sequence is unknown or variable). This sequence is usually seen in humans and other primates [1], whereas dc is usually last to emerge in chimpanzees [14,15]. The state of deciduous and permanent tooth formation (electronic supplementary material, figure S1 and table S1) corresponds to an age of around 2 years based on either chimpanzee or human standards, although there is greater similarity with humans (figure 2). In contrast to U.W. 101-1400 and humans, chimpanzees at a comparable stage of M_1_ formation have some of the P_4_ crown formed and less developed canines [12,16,17].

### (b) U.W. 101-377

Premolars and M_1_ are in occlusion, while C and M_2_ have attained only gingival emergence. Greater apical wear on the canine indicates an emergence sequence of [M_1_–I_1_–I_2_]–[P_3_–P_4_]–[C–M_2_]–M_3_ (electronic supplementary material, figure S2). The premolar–M_2_ sequence matches the most common pattern among humans, which is much less common in chimpanzees and unknown in Early–Middle Pleistocene hominins [18]. By contrast, relatively late canine emergence is most common among chimpanzees and Plio-Pleistocene hominins, but rarer in humans.

U.W. 101-377 tooth formation cannot be pigeonholed into ‘human’ or ‘chimpanzee’ categories. Compared with humans, C is underdeveloped relative to P_3-4_ and M_2_ (figure 2; electronic supplementary material, figure S3 and table S2). Compared with chimpanzees, all teeth are underdeveloped relative to M_1_, while C and P_3_ are advanced over P_4_ and M_2_. M_2_ development at gingival emergence, with about half the root formed, is similar to apes [19], whereas human M_2_s usually attain gingival emergence with around 75% root length [20]. Thus, U.W. 101-377 juxtaposes human-like M_2_ emergence sequence with chimpanzee-like root development.

## 4. Discussion

These mandibles capture two snapshots in the dynamic processes of dental development in *H. naledi*. Although many other immature dental specimens are known for this species, U.W. 101-1400 and -377 provide the only secure associations of multiple teeth. Future discoveries in Rising Star Cave will demonstrate whether these are representative of the species. As with the rest of the species' skeleton, tooth formation and emergence show a mix of primitive and derived features. Many features are probably plesiomorphies at various levels. For instance, because dc emergence usually precedes dp4 among anthropoids [1] including early hominins [20], this may be the ancestral condition for all apes and monkeys. The relatively underdeveloped M_2_ roots at emergence are also seen in apes and fossil hominins [21], implying a hominine symplesiomorphy. Underdeveloped C crowns compared to chimpanzees at the same state of M_1_ formation as U.W. 101-1400 may reflect hominins’ reduced canine size [6].

The similarity in development of U.W. 101-1400 to humans may be a function of its young age. This infant shows comparable states of permanent tooth formation to *A. robustus* infants SK 64, -438 and -3978 [6,21], and is only slightly less mature than *H. antecessor* specimen ATD6-112 [22]. The significance of this similarity among morphologically distinct hominins is unclear: *A. robustus* shows a prolonged period of deciduous tooth emergence similar to humans [23], but also formed its permanent teeth more rapidly than humans [3]. By contrast, developing M1s from ATD6 provide the earliest evidence of a human-like delayed M1 emergence. The state of tooth formation in the *H. naledi* infant may therefore reflect its being an infant hominin rather than bear any life history significance.

More intriguing is the developmental status of U.W. 101-377, with an emerging M_2_ and fully emerged premolars. All other Plio-Pleistocene hominin taxa for which this sequence can be discerned (South African australopiths, *H. erectus* and Neandertals) emerged M_2_ before one or both of the premolars [4,18,24,25]. This sequence also characterizes apes, and may reflect relatively rapid craniofacial and somatic growth [1,2,6,26]. That *H. naledi* displays the pattern most common among humans may indicate slower growth in this species. Brain size as small as that of *H. naledi* [7], however, is associated with more rapid growth and maturation among primates [26]. The occurrence of the human-like sequence in *H. naledi* and a primitive sequence in other hominins raises questions about the adaptive significance of tooth emergence sequences. Future research may shed light on these questions: tooth histological data may yield accurate chronological estimates of age at death for these specimens (e.g. [4]), demonstrating the actual pace of life history in *H. naledi*. Further work should also examine the degree to which posterior molar emergence is constrained by facial size and growth [2,26].

## Supplementary Material

Electronic Supplementary Material for Dental development in Homo naledi

## Data accessibility

Tooth formation states and original images are in the electronic supplementary material.
