# Supplementary material for: Dental development in Homo naledi
Source: Biol Lett. 2017 Aug 30;13(8):20170339. doi: 10.1098/rsbl.2017.0339 (PMC5582112; doi:10.1098/rsbl.2017.0339)
Supplement: Electronic Supplementary Material for Dental development in Homo naledi [file rsbl20170339supp1.doc]

Electronic Supplementary Material for

Dental development in *Homo naledi*

Zachary Cofran and Christopher S. Walker

**­­­S1. Preservation, association & development**

U.W. 101-1400 is a left hemi-mandible corresponding to Dinaledi Hominin 6 [1]. The dc, dp_3_, dp_4_, and M_1_ are *in situ*. Anteriorly, the buccal/labial corpus is broken away, exposing the dc root and crypts for I_1-2_. The LC crypt is at the very inferior-most corpus, and the developing crown is exposed. The I_2_ crypt wall follows the contour of post-incisive plane and terminates just below the posterior-most extent of the plane, though this does not elucidate the state of development of this tooth. Posteriorly, the fragment is missing all bone distal to the M_1_ crypt, obfuscating the developmental status of M_2_.

The deciduous canine and molars are fully erupted, and the developing permanent C, P_3_ and M_1_ crowns are visible in their crypts (Fig. 1 and Supplementary Figure S1). All dm_1_ cusps are worn except the metaconid, dc has only slight apical wear, and dm_2_ is unworn except for a small facet on the protoconid. Preserved deciduous teeth are mostly complete (Table S1; Fig. S1). The dc root is broken distally, but appears to have reached full length, if not apical closure. The dm1 roots are complete with closed apices but root resorption had not begun. The dm_2_ roots have reached complete length but the apices are open. The C crown height is about 6.6 mm and the distal tubercle is not yet formed, equating to ~50% complete compared with adult Cs (Supplementary Table S3). P_3_ trigonid cusps are just barely coalesced. The P_4_ crypt had begun to form beneath dp_4_, but there is no indication of P_4_ mineralization; the crypt is not exposed so it is highly unlikely that the crown began formation and was subsequently lost. The M_1_ crown is nearly complete with no indication of root development. Measures of tooth crown height are very close to those of the largely unworn M_1_ of U.W. 101-377 (i.e., <1 mm difference). Thus, while the crown is not fully complete, it is closest to this stage and scored as such in the analysis.

U.W. 101-377 is a right hemi-mandible from the distal C alveolus to the mesial M_3_ crypt, and corresponds to a paratype of this species [2]. Gingival emergence of the C and M_2_ is indicated by a flattened wear facet along the distal edge of C and a small facet on the protoconid of an otherwise unworn M_2_ (Fig. S2). The mandible is broken distally exposing the M_3_ crypt, the preservation of which obfuscates the tooth’s state of development. M_1_ crown and roots are completely formed, and P_3_ roots are nearly complete with root apices barely open, C and P4 roots are around 75% complete, and M_2_ roots are about 50% complete (Table S3).

**S2. Assessing tooth development and estimating age**

We scored the state of development of each tooth according to both the Demirjian [3,4] and Moorrees [5,6] standards, based on microCT sections through all teeth (Supplementary Tables S1-2). We measured root lengths from the cemento-enamel junction (CEJ) to their apical-most extent, along their sagittal midlines and buccal roots (the buccal roots are the longest of multi-rooted premolars and molars). The same measurements were taken on adult mandibular teeth for a quantitative assessment of relative root development (Table S3). Where roots were affected distally by abrasion, the measurement was taken from the CEJ to the bony alveolar surface beneath the developing root, providing a conservative maximum length. ZDC and CSW scored each dentition independently, and results were usually identical and never differed by more than one stage. We are therefore confident in our assessments of development.

To determine whether patterns of tooth formation in *H. naledi* are more similar to humans or chimpanzees, we used published standards to provide age estimates of individual teeth for each specimen. Human female and male standards [5,6] produce the same results so here we present only comparisons with females. Chimpanzee standards are from a captive population [4] combining sexes due to small sample sizes; canine maturation is based on female maturation standards only, however, since their canines are significantly smaller, and therefore more hominized, than males’. As it has been suggested that wild chimpanzees and captive chimpanzees display different dental maturation [7], wild chimpanzees of known age [8] are also included.

As noted in the main text, U.W. 101-377 indicates M_2_ emergence after the premolars. Corroborating this pattern, the U.W. 101-1283 holotype mandible, a young adult, presents P_3-4_ with small dots of dentin exposure, while M_2_ has no exposure.

Table S1. Tooth formation of U.W. 101-1400

| Tooth | Moorrees | Demirjian |
| --- | --- | --- |
| dc | Rc+ | 7+ |
| dp3 | Ac | 8 |
| dp4 | Rc | 7 |
| M1 | Crc | 4 |
| C | Cr1/2 | 3 |
| P3 | Cco | 2 |
| P4 | 0 | 0 |

Tooth formation stages for individual teeth based on both Moorrees et al. [5] and Demirjian et al. [3] standards. 0 indicates tooth formation had not yet begun.

**Table S2**. Tooth formation of U.W. 101-377

| Tooth | Moorrees | Demirjian |
| --- | --- | --- |
| C | R3/4 | 6 |
| P3 | A1/2 | 7 |
| P4 | R3/4 | 6 |
| M1 | Ac | 8 |
| M2 | R1/2 | 6 |

Tooth formation stages for individual teeth based on both Moorrees et al. [5] and Demirjian et al. [3] standards.

**Table S3. *Homo naledi* crown heights and root lengths**

| **Specimen ID (UW 101-)** | **Tooth** | **Measurement** | **Length (mm)** | **Source** | **Note** |
| --- | --- | --- | --- | --- | --- |
| 1400 | C | crown (labial) | 6.6 | CT, 3D mesh | Crown unworn |
| 1076 | C | crown (labial) | 12.5 | CT | Very mild apical wear |
| 1126 | C | crown (labial) | 13.7 | CT | Crown unworn |
| 339 | C | crown (labial) | 12.9 | CT | Crown unworn |
| 377 | C | crown (labial) | 11.4 | 3D mesh | Very mild apical wear |
| 886 | C | crown (labial) | 13.7 | CT | Crown unworn |
| 985 | C | crown (labial) | 14.6 | CT | Crown unworn |
|  | Complete average: | | 13.1 |  |  |
|  | 1400 % complete: | | 51% |  |  |
|  |  |  |  |  |  |
| 377 | C | root (mesial) | 14.1 | 3D mesh | Taken to abraded root end |
| 377 | C | root (mesial) | 14.6 | 3D mesh | Taken from bony floor of alveolus |
| 010 | C | root (mesial) | 20.5 | CT |  |
| 1283 | C | root (mesial) | 20.2 | CT |  |
|  | Complete average: | | 20.4 |  |  |
|  |  | 377 % complete: | 69% |  | Taken to abraded root end |
|  |  | 377 % complete: | 72% |  | Taken from bony floor of alveolus |
|  |  |  |  |  |  |
| 377 | P4 | root (mesial) | 13.3 | 3D mesh, CT | Taken from bony floor of alveolus |
| 001 | P4 | root (mesial) | 15.9 | CT |  |
| 1283 | P4 | root (mesial) | 16.0 | CT |  |
|  | Complete average: | | 16.0 |  |  |
|  |  | 377 % complete: | 83% |  |  |
|  |  |  |  |  |  |
| 377 | M2 | root (mesiobuccal) | 8.2 | 3D mesh | Taken from bony floor of alveolus |
| 001 | M2 | root (mesiobuccal) | 15.7 | 3D mesh |  |
| 1283 | M2 | root (mesiobuccal) | 15.6 | 3D mesh |  |
|  | Complete average: | | 15.7 |  |  |
|  |  | 377 % complete: | 52% |  |  |

Permanent canine crown heights and canine, P_4_ and M_2_ root lengths were measured on 2D microCT images and/or 3D surface meshes. This sample includes the most complete dental specimens in the Dinaledi chamber.

**Figure S1. MicroCT slices of individual teeth of U.W. 101-1400**

**
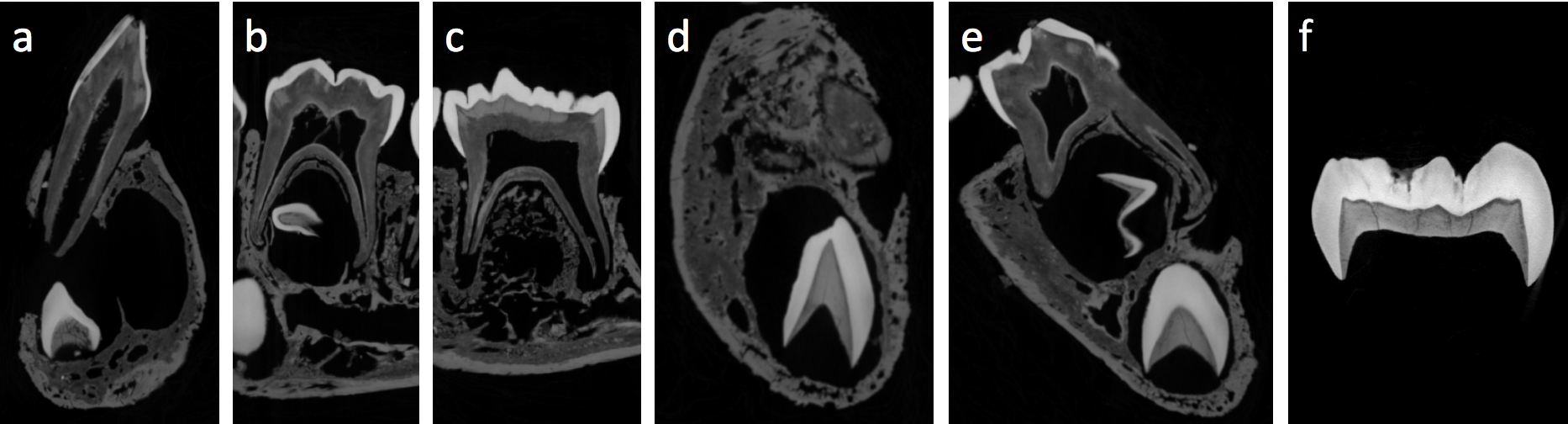
**

Sections through the developing teeth of U.W. 101-1400. a-f: dc, dp_3_, dp_4_, C, P_3_, M_1_. Mesial is to the left in b, c and f. Deciduous premolar and permanent molar sections (b, c, f) are mesiodistal, while canine and premolar sections (a, d, e) are labio-/buccolingual. Note the empty, developing crypt for P4 beneath the dp_4_ (c). Images are to the same scale.

**Figure S2. Occlusal wear on the U.W. 101-377 C and M_2_**

**
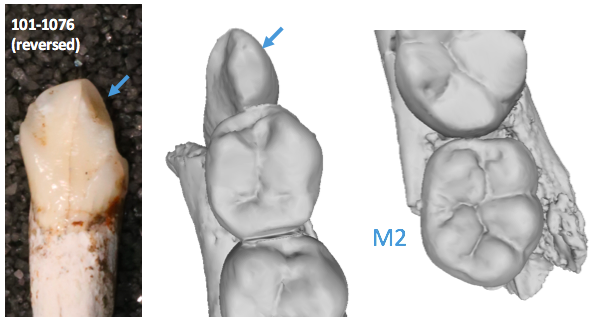
**

Occlusal wear in the U.W. 101-377 canine and M_2_. U.W. 101-1076 (left) is the probable antimere of the 101-377 canine (center), based on size, shape, wear, and root development. Note the strong, matching wear facets on the canines, but the virtually unworn M_2_. Images not to scale.

**Figure S3. MicroCT slices of individual teeth of U.W. 101-377**


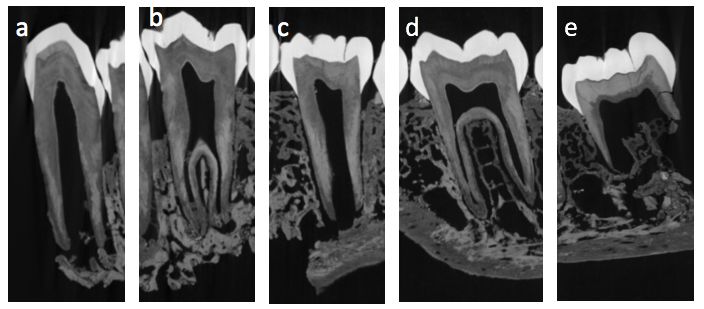


Mesiodistal sections through the developing teeth of U.W. 101-377. a-e: C, P_3_, P_4_, M_1_, M_2_. Mesial is to the left. Note the open P_3_ root apices (b). Images are to the same scale.

**References**

1. Laird, M. F. et al. 2017 The skull of *Homo naledi*. *J. Hum. Evol.* **104**, 100–123. (doi:10.1016/j.jhevol.2016.09.009)

2. Berger, L. R. et al. 2015 *Homo naledi*, a new species of the genus *Homo* from the Dinaledi Chamber, South Africa. *eLife* **4**, e09560. (doi:10.7554/eLife.09560)

3. Demirjian, A., Goldstein, H. & Tanner, J. M. 1973 A new system of dental age assessment. *Hum. Biol.* **45**, 211–227. (doi:10.2307/41459864)

4. Kuykendall, K. L. 1996 Dental development in chimpanzees (*Pan troglodytes*): The timing of tooth calcification stages. *Am. J. Phys. Anthropol.* **99**, 135–157. (doi:10.1002/(SICI)1096-8644(199601)99:1<135::AID-AJPA8>3.0.CO;2-#)

5. Moorrees, C. F., Fanning, E. A. & Hunt, E. E. 1963 Age Variation of Formation Stages for Ten Permanent Teeth. *J. Dent. Res.* **42**, 1490–1502. (doi:10.1177/00220345630420062701)

6. Shackelford, L. L., Harris, A. E. S. & Konigsberg, L. W. 2012 Estimating the distribution of probable age-at-death from dental remains of immature human fossils. *Am. J. Phys. Anthropol.* **147**, 227–253. (doi:10.1002/ajpa.21639)

7. Zihlman, A., Bolter, D. & Boesch, C. 2004 Wild chimpanzee dentition and its implications for assessing life history in immature hominin fossils. *Proc. Natl. Acad. Sci.* **101**, 10541–3. (doi:10.1073/pnas.0402635101)

8. Smith, T. M., Smith, B. H., Reid, D. J., Siedel, H., Vigilant, L., Hublin, J. J. & Boesch, C. 2010 Dental development of the Taï Forest chimpanzees revisited. *J. Hum. Evol.* **58**, 363–373. (doi:10.1016/j.jhevol.2010.02.008)
